# Inhibition of the cGAS‑STING Pathway Reduces Cisplatin-Induced Inner Ear Hair Cell Damage

**DOI:** 10.1007/s12264-024-01334-8

**Published:** 2024-12-16

**Authors:** Ying Sun, Shengyu Zou, Xiaoxiang Xu, Shan Xu, Haiying Sun, Mingliang Tang, Weijia Kong, Xiong Chen, Zuhong He

**Affiliations:** 1 Department of Otorhinolaryngology-Head and Neck Surgery, Zhongnan Hospital of Wuhan University, Wuhan, 430071 China; 2 State Key Laboratory of Digital Medical Engineering, Department of Otolaryngology Head and Neck Surgery, Zhongda Hospital, School of Life Sciences and Technology, Advanced Institute for Life and Health, Jiangsu Province High-Tech Key Laboratory for Bio-Medical Research, Southeast University, Nanjing, 210096 China; 3 Department of Otolaryngology, The First Hospital of China Medical University, Shenyang, 110001 China; 4 Department of Otorhinolaryngology, Union Hospital, Tongji Medical College, Huazhong University of Science and Technology, Wuhan, 430022 China; 5 Institute for Cardiovascular Science and Department of Cardiovascular Surgery of the First Affiliated Hospital, Suzhou Medical College of Soochow University, Soochow University, Suzhou, 215000 China

**Keywords:** Ototoxicity, Hearing loss, cGAS-STING, Hair cell, Mitochondrial dysfunction

## Abstract

Although cisplatin is a widely used chemotherapeutic agent, it is severely toxic and causes irreversible hearing loss, restricting its application in clinical settings. This study aimed to determine the molecular mechanism underlying cisplatin-induced ototoxicity. Here, we established *in vitro* and *in vivo* ototoxicity models of cisplatin-induced hair cell loss, and our results showed that reducing STING levels decreased inflammatory factor expression and hair cell death. In addition, we found that cisplatin-induced mitochondrial dysfunction was accompanied by cytosolic DNA, which may act as a critical linker between the cyclic GMP-AMP synthesis−stimulator of interferon genes (cGAS-STING) pathway and the pathogenesis of cisplatin-induced hearing loss. H-151, a specific inhibitor of STING, reduced hair cell damage and ameliorated the hearing loss caused by cisplatin *in vivo*. This study underscores the role of cGAS-STING in cisplatin ototoxicity and presents H-151 as a promising therapeutic for hearing loss.

**Supplementary Information:**

The online version contains supplementary material available at 10.1007/s12264-024-01334-8.

## Introduction

Cisplatin is a commonly used chemotherapeutic drug and is widely used to treat a variety of malignant cancers [1, 2]. However, during treatment, cisplatin can cause severe adverse off-target effects (e.g., ototoxicity, neurotoxicity, nephrotoxicity, and cardiometabolic abnormalities) [3], resulting in poor quality of life. Cisplatin-induced ototoxicity can cause irreversible sensorineural hearing loss by severely damaging outer hair cells (OHCs) and other structures in the inner ear, limiting the clinical application of this drug [4–6]. The exact pathogenesis of ototoxicity is largely unclear. Possible mechanisms include increased inflammation, reactive oxygen species (ROS) production, and apoptosis [7, 8]. Inflammation seems to be the first event occurring in cochlear hair cells after cisplatin treatment, but the molecular basis of the inflammatory phenotype of cisplatin-induced hearing loss remains unclear.

Cyclic GMP-AMP synthase (cGAS) is considered an important DNA sensor that detects damaged DNA in the cytoplasm. When cells are activated by nociceptive stimuli, cGAS recognizes and binds cytoplasmic DNA, after which it undergoes a conformational change to generate a second messenger molecule, cyclic guanosine monophosphate (GMP)-adenosine monophosphate (AMP) (cGAMP). Then, cGAMP activates the stimulator of interferon genes (STING) and induces its translocation from the endoplasmic reticulum to the Golgi [9]. Subsequently, activated STING binds and phosphorylates TANK-binding kinase 1 (TBK1) and IκB kinase, which facilitates the translocation of nuclear factor κB (NF-κB) to the nucleus, leading to activation of the NF-κB signaling pathway and the production of various inflammatory factors [10]. However, increasing evidence indicates that the activation of the cGAS-STING signaling pathway is a key factor contributing to the inflammatory phenotype in diseases [11–13]. Maekawa *et al*. [14] reported that the cGAS-STING pathway plays an important role in cisplatin-induced acute kidney injury. Moreover, inhibition of STING activation attenuates aging-related inflammation in multiple peripheral organs in mice and leads to improvements in tissue function [15]. Although numerous studies have described the importance of the cGAS-STING pathway in various pathological processes, whether it is involved in OHC death caused by cisplatin is currently unknown.

Here, we established an HC damage model and assessed the activation of cGAS/STING and its downstream targets during cisplatin treatment. Further analysis of the cisplatin-induced ototoxic cell model showed that STING knockdown in HEI-OC1 cells can block inflammation and cell death. In addition, our novel findings suggest that cytosolic mtDNA participates in cisplatin-induced HC injury, indicating that mtDNA may be an activator of the cGAS-STING pathway. Finally, we found that the STING-specific inhibitor H-151 reduced cisplatin-induced OHC damage and hearing loss in mice. These data support the essential role of the cGAS-STING pathway in mediating cisplatin-induced ototoxicity.

## Materials and Methods

### Animals

Six- to eight-week-old male FVB/NJ mice with normal hearing confirmed by the auditory brainstem response (ABR) were used. The mice were housed under standard laboratory conditions on a 12 h:12 h light-dark cycle at a constant temperature (22–24°C) and relative humidity of 50%–60% with free access to water and rodent chow. The protocols were consistent with the National Institutes of Health Guide for the Care and Use of Laboratory Animals. All animal experiments were approved by the Laboratory Animal Welfare & Ethics Committee of Zhongnan Hospital of Wuhan University.

Three groups of mice (control, cisplatin, and H-151 + cisplatin) were used. As in previous studies [16], the mice in the cisplatin group were administered furosemide and cisplatin to establish an acute ototoxicity model. First, 200 mg/kg furosemide (South Land Pharmaceutical, Guangdong, China) was injected intraperitoneally. Half an hour later, 2 mg/kg cisplatin (HY-17394, MedChemExpress, New Jersey, USA) was administered subcutaneously. One hour later, 1 mL of isotonic NaCl was injected intraperitoneally. In the H-151 + cisplatin group, 10 mg/kg H-151 (HY-112693, MedChemExpress) was injected intraperitoneally one day before the first furosemide injection and 1 h before each furosemide injection to test the protective effect of H-151 *in vivo*. The control group was injected with an equal volume of saline. After the final injection, the mice were allowed to recover for three days before cisplatin treatment. ABR tests were applied to detect changes in hearing.

### ABR Test

The ABR test is an objective measurement of the hearing threshold. Briefly, the mice were anesthetized *via* an intraperitoneal injection of 50 mg/kg pentobarbital sodium and kept warm at 37°C with a thermostatic heating pad during the ABR recordings. After the mice were fully anesthetized, electrodes were subcutaneously inserted behind the tested ear, behind the contralateral ear, and at the middle of the head. The hearing threshold was assessed at three frequencies (8, 16, and 32 kHz) on a TDT System III apparatus (Tucker Davies Technologies, Gainesville, FL, USA). Each frequency was measured from 90 dB and lowered by 10 dB each time until no ABR wave II response was detected to determine the threshold for each frequency.

### Immunofluorescence Staining of Cochlear Surface Preparations

In accordance with the protocol detailed by Fang and colleagues [17], we proceeded as follows: temporal bones were extracted and locally infused with a solution of 4% paraformaldehyde in phosphate-buffered saline (PBS), pH 7.4, through the round and oval windows and then incubated with this fixative at 4°C overnight. The cochlear samples were washed in PBS a minimum of three times for 5–10 min each throughout the various stages. Following 3 days of decalcification in a 4% solution of sodium ethylenediaminetetraacetic acid (in PBS, pH 7.4) at 4 °C, the cochleae were cut into apex, middle, and base segments. These segments were subsequently mounted on 10-mm circular coverslips using Cell-Tak adhesive (#354240, BD Biosciences, New Jersey, USA). Next, the samples were permeabilized in 2% Triton X-100 for 15 min and then blocked with 10% normal donkey serum for 30 min at room temperature, followed by incubation with a primary polyclonal rabbit anti-myosin 7a antibody (#25–6790, Proteus Bioscience, Waltham, USA) at 1:500 dilution overnight at 4°C. The samples were subsequently incubated with Alexa Fluor 488-conjugated secondary antibodies (1:500, A-11008, Thermo Fisher Scientific, Waltham, USA), phalloidin (1:500, 21836, Thermo Fisher Scientific), and 4',6-diamidino-2-phenylindole dihydrochloride (DAPI) overnight at 4°C in the dark.

Following three washes with PBS, 8 μL of mounting medium (S3023, DAKO, Copenhagen, Denmark) was added to the samples. These samples were then covered with an additional round coverslip and mounted on microscope slides. Finally, the edges of the coverslips were securely sealed using nail polish. Images of the immunostained samples were acquired under a Zeiss LSM 900 microscope at 40× magnification, ensuring consistent Z-stack conditions for all images.

### HC Counts

Images of the entire stretch from the apex to the base of the cochlea, highlighting myosin 7a-labeled and phalloidin-stained surfaces, were captured under a Zeiss microscope with a 10× lens. The lengths of the cochlear epithelia were measured and recorded in millimeters. Given the negligible loss of inner HCs under our cisplatin treatment conditions, the focus was on counting OHCs from the apex to the base across the full extent of the cochlear spiral. OHCs were counted from the apex to the base along the entire length of the cochlear epithelium. The percentage of HC loss in each 0.5-mm length of the epithelium was plotted as a function of the cochlear length in a cytocochleogram [18].

### Cell Culture

HEI-OC1 is a mouse auditory cell line available for research purposes [19]**.** HEI-OC1 cells were cultured in a 33°C incubator with 5% CO_2_ in Dulbecco’s modified Eagle’s medium (DMEM; Thermo Fisher Scientific) supplemented with 10% fetal bovine serum (F8318, Sigma, Darmstadt, Germany) without antibiotics. Cultured cells were subcultured with 0.25% trypsin/EDTA (Thermo Fisher Scientific) after reaching 80% confluence.

### Stable Cell Line Generation

Stable STING-EGFP-expressing HEI-OC1 cells were generated by transduction with the pcSLenti-CMV-Tmem173-linker-EGFP-3×FLAG-PGK-puro-WPRE3 lentivirus (OBiO Technology, Shanghai, China). Briefly, 2 × 10^5^ cells were incubated in a virus-containing culture medium (MOI = 40:1) and polybrene (10 µg/mL) for 24 h. Then, the virus-containing medium was removed. After an additional 48 h of incubation, HEI-OC1 cells stably expressing STING through lentivirus transduction were selected with 10 μg/mL puromycin (Yeasen, Shanghai, China). After this, stable STING-EGFP-expressing HEI-OC1 cells were washed twice in PBS, incubated with 4% paraformaldehyde for 10 min at room temperature, permeabilized in 0.5% Triton X-100 for 10 min, blocked for 1 h with 10% donkey serum, and then incubated with an anti-GM130 antibody (1:500, 610822, BD Biosciences) overnight at 4°C in the dark. The next day, the cells were washed three times in PBS and incubated with an Alexa Fluor 594-conjugated secondary antibody (1:500, A-11012, Thermo Fisher Scientific) and DAPI overnight at 4°C in the dark. The coverslips were mounted on slides with a drop of fluorescence mounting medium (Dako, S3023) and sealed with clear nail polish. Labeling patterns were observed under a confocal laser scanning microscope (Leica TCS SP8, Leica Microsystems GmbH, Wetzlar, Germany).

### Transfection

siRNA-STING was designed and synthesized by OBiO Technology (Shanghai, China) to inhibit STING expression. HEI-OC1 cells were cultured on plates for 24 h and transfected with the STING or control siRNA using the Lipofectamine RNAiMAX reagent (13778150, Thermo Fisher Scientific). The transfected cells were exposed to cisplatin or a control medium, incubated for 24 h, and harvested for analysis. The following siRNAs were used to knock down STING expression: sense 5′-GGAUCCGAAUGUUCAAUCAGCTT-3′ and antisense 5′-GCUGAUUGAACAUUCGGAUCCTT-3′.

### RNA-Seq

HEI-OC1 cells maintained for 24 h in a culture medium served as the control group and those cultured for 24 h in 30 μmol/L cisplatin served as the treatment group; the cells were subjected to transcriptome sequencing at Wefindbio Biotechnology Co., Ltd. (Wuhan, China) (three independent replicates per group).

### Quantitative Real-time PCR

Total RNA was extracted from cells using TRIzol reagent (R411-01, Vazyme, Nanjing, China) according to the manufacturer’s protocol, and complementary DNA (cDNA) was synthesized from the purified RNA using HiScript III RT SuperMix for qPCR (+gDNA wiper) (R323, Vazyme). Quantitative RT‒PCR was applied using Hieff UNICON Universal Blue qPCR SYBR Green Master Mix (11184, Yeasen, Shanghai, China) on a StepOnePlus Real-Time PCR System (Applied Biosystems, USA). ACTIN was used as a housekeeping gene for normalization, and the 2^−ΔΔCt^ method was used to assess relative gene expression. The sequences of the primers used are listed in Table 1.

**Table 1:** Primers for real-time PCR.

Gene	Species	Forward primer	Reverse primer
*Ccl7*	Mouse	TCACTCTCTTTCTCCACCATGAG	CAGACTTCCATGCCCTTCTTTGT
*Ifi202b*	Mouse	GAAAGGCTGGTTGATGGAGAGTT	TGTCCAGATACCACCACTTTCAT
*Il20rb*	Mouse	TCATGCTGATTCTCGTGGTTGTA	AGCTTCTGAGACGAACTGGTTATT
*Tnfrsf12a*	Mouse	GCTCTTAGTCTGGTCCTGGTTT	CCTCTCCACCAGTCTCCTCTATG
*Tnfrsf13c*	Mouse	CCCACCCAGTGCAATCAGAC	AGGCTGCTTGTATGTCCAGT
*Tnfsf18*	Mouse	GTTGCTCTGTTCTTTGGGTACAC	AGCTTCCCATCAGATGTCGTATT
*Ifna4*	Mouse	CAGAGAGTGACCAGCATCTACAA	GGTTATAAGTGTGAGGCAGGTCA
*Cxcl1*	Mouse	CCGAAGTCATAGCCACACTCAAG	ACCAGACAGGTGCCATCAGAG
*Cxcl10*	Mouse	GTGTTGAGATCATTGCCACGATG	TCAGAAGACCAAGGGCAATTAGG
*Ccl2*	Mouse	GGTGTCCCAAAGAAGCTGTAGTT	AGCTTCAGATTTACGGGTCAACT
*Sting*	Mouse	GGGTTTATTCCAACAGCGTCTAC	TAGACAATGAGGCGGCAGTTATT
*Actin*	Mouse	GGCTGTATTCCCCTCCATCG	CCAGTTGGTAACAATGCCATGT

### Western Blot

The samples were lysed in RIPA buffer (P0013B, Beyotime, Shanghai, China) supplemented with phenylmethylsulfonyl fluoride, phosphatase inhibitor (4906837001, Roche, Basel, Switzerland), phosphatase inhibitor cocktail 2 (P5726, Roche), and phosphatase inhibitor cocktail 3 (P0044, Roche). A BCA protein quantification kit (P0010, Beyotime) was used to measure the total protein concentration. Protein samples (15 µg per lane) were separated *via* sodium dodecyl sulfate-polyacrylamide gel electrophoresis and transferred to polyvinylidene fluoride membranes (Millipore, Darmstadt, Germany). The membranes were blocked with 5% nonfat milk for 1 h at room temperature and then incubated overnight at 4°C with primary antibodies. The primary antibodies used were anti-STING (1:1000; ab288157, Abcam, Cambridge, UK); anti-cGAS (1:1000; #31659, Cell Signaling Technology, Danvers, MA, USA); anti-TBK1 (1:1000; #38066, Cell Signaling Technology); anti-phospho-TBK1 (Ser172) (1:1000; #5483, Cell Signaling Technology); anti-phospho-NF-κB p65 (Ser536) (1:1000, #3033, Cell Signaling Technology); anti-NF-κB p65 (1:1000, #4764, Cell Signaling Technology); and anti-β-actin (1:1000, 20536-1-AP, ProteinTech, Wuhan, China). The membranes were then incubated with anti-rabbit secondary antibodies (ANT020, Antgene, Wuhan, China) for 1 h at room temperature. Finally, a chemiluminescence kit (E423, Vazyme) was used to detect the signals.

### Flow Cytometry for Apoptosis Detection

HEI-OC1 cells were stained using a FITC Annexin V Apoptosis Detection Kit (556547, BD Biosciences). Annexin V was used to analyze apoptosis, and PI was used to differentiate between live and dead cells. The samples were trypsinized and collected *via* centrifugation at 1,000 rpm for 5 min, washed with PBS, and resuspended in binding buffer. Then, Annexin V and PI were added, and the mixture was gently vortexed and incubated for 15 min in the dark at room temperature. Flow CytExpert (CytoFLEX, Beckman, Brea, CA, USA) was promptly used to analyze the samples.

### Flow Cytometry for Mitochondrial Membrane Potential Measurement

Image-iT™ TMRM Reagent (I34361, Thermo Fisher Scientific) was used as an indicator of the mitochondrial membrane potential. After the HEI-OC1 cells were collected, they were washed with PBS, incubated for 30 min at 37°C in the dark, and analyzed *via* CytExpert (CytoFLEX, Beckman).

### Mitochondrial ROS Assay

MitoSOX Red (M36008, Thermo Fisher Scientific) was used to analyze mitochondrial ROS production. First, we measured mitochondrial ROS levels using flow cytometry. After trypsinization, the HEI-OC1 cells were collected *via* centrifugation and washed with PBS. The cell pellets were then resuspended in a solution containing MitoSOX Red for 15 min at 37°C in the dark and analyzed *via* CytExpert (CytoFLEX, Beckman). In addition, we assessed mitochondrial ROS levels *via* fluorescence staining. After treatment and washing with PBS, the cells were stained with MitoSOX Red in prewarmed serum-free DMEM in the dark for 30 min in an incubator. Images were acquired under a Zeiss LSM 900 confocal microscope.

### Transmission Electron Microscopy

HEI-OC1 cells were fixed in 2.5% glutaraldehyde (Sigma‒Aldrich) for 24 h at 4°C in the dark and 1% osmic acid (Sigma‒Aldrich) for 1–2 h, dehydrated with acetone (Sinopharm Chemical Reagent), and embedded in Araldite CY212 (TAAB). Ultrathin sections were stained with uranyl acetate in alcohol (Polysciences) and alkaline lead citrate (Sigma‒Aldrich). The sections were gently washed with distilled water and examined under a Hitachi HT 7800 transmission electron microscope (Hitachi Ltd, Tokyo, Japan) by Servicebio Technology (Wuhan, China).

### Immunofluorescence Staining for Mitochondrial DNA Release

The method used to detect mitochondrial DNA release was adapted from previously published methods [20, 21]. HEI-OC1 cells were incubated with the indicated treatments, washed twice with PBS, incubated with 4% paraformaldehyde for 10 min at room temperature, permeabilized with 0.5% Triton X-100 for 10 min, blocked for 1 h with 10% donkey serum, and then incubated with an anti-dsDNA antibody (1:200, 690014, Progen, Heidelberg, Germany) and an anti-TOM20 antibody (1:500, 11802-1-AP, ProteinTech) overnight at 4°C. The following day, the cells were washed three times with PBS and incubated with an Alexa Fluor 488-conjugated secondary antibody (1:500, Thermo Fisher, A-11001), Alexa Fluor 594-conjugated secondary antibody (1:500, Thermo Fisher, A-11012), and DAPI overnight at 4°C in the dark. The coverslips were mounted on slides with a drop of fluorescence mounting medium (S3023, Dako) and sealed with clear nail polish. Labeling patterns were examined under a Zeiss LSM880 with an Airyscan confocal microscope. The number of DNA foci outside the nucleus and the mitochondrial perimeter were measured manually to quantify the number of cytosolic DNA foci.

### TUNEL Assay

The TUNEL assay was applied according to the protocols of the In Situ Cell Death Detection Kit TMR Red (1215679291, Roche). After staining with DAPI and the required antibodies, a confocal laser scanning microscope (Leica TCS SP8) was used for visualization.

### Statistical Analysis

All of the data are presented as the mean ± SD. All of the experiments were repeated at least three times. Statistics were calculated using Microsoft Excel and GraphPad Prism 9. Statistical significance was determined using a two-tailed unpaired *t* test when two groups were compared and using one-way ANOVA and Dunnett's multiple comparisons test when more than two groups were compared. *P* values <0.05 were considered to indicate statistical significance.

## Results

### The Survival of HEI-OC1 Cells Decreases as the Cisplatin Concentration Increases

**Fig. 1:**
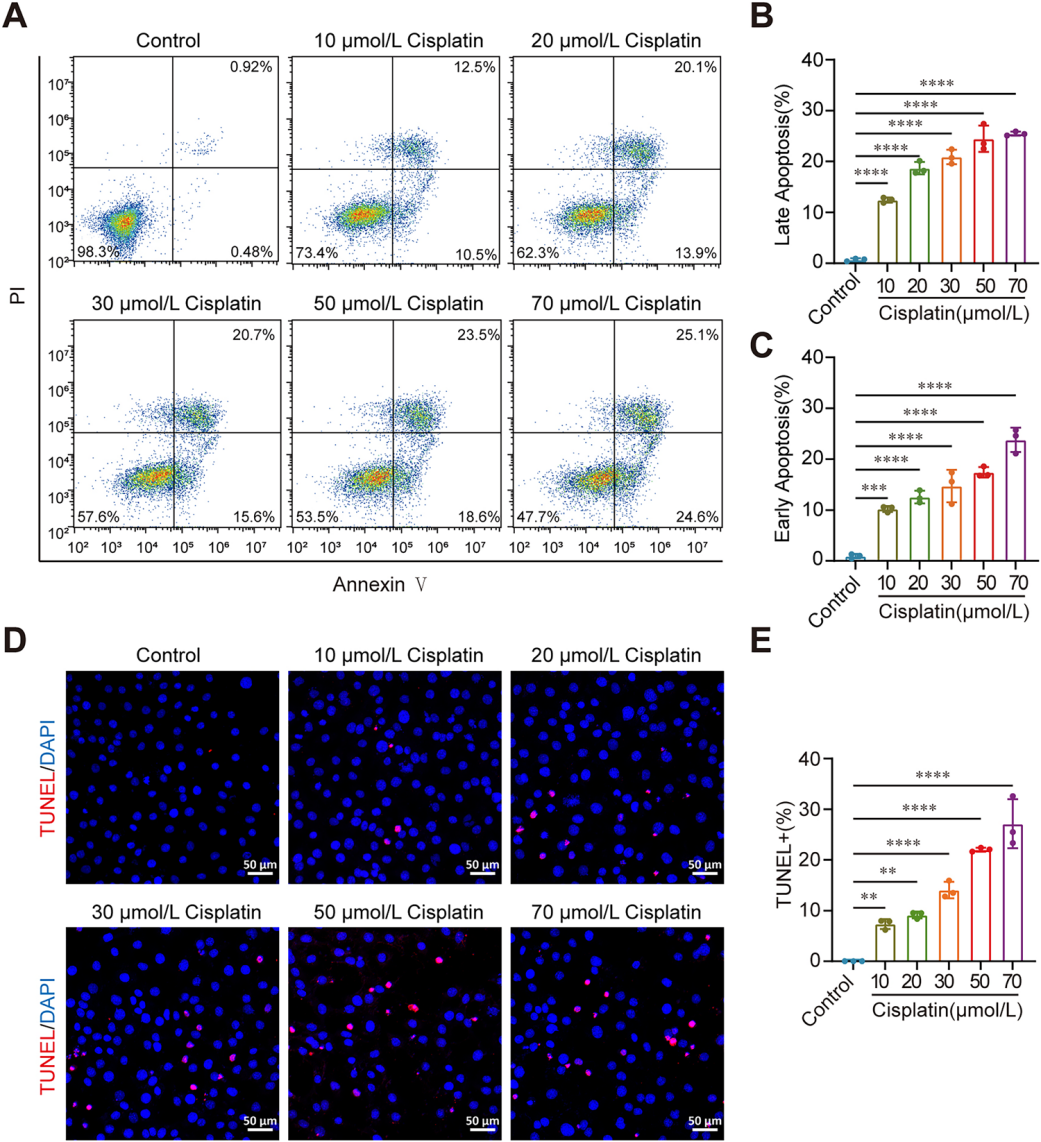
Cisplatin causes HEI-OC1 cell damage. **A** Flow cytometry analysis of the effect of cisplatin on cell apoptosis. Apoptotic cells were detected with an Annexin V-FITC/PI kit. **B** Quantification of the percentage of late apoptotic cells in A (*n* = 3, ANOVA). **C** Quantification of the percentage of early apoptotic cells in A (*n* = 3, ANOVA). **D** Representative images of TUNEL staining of HEI-OC1 cells. **E** Quantification of TUNEL-positive cells (*n* = 3, ANOVA). TUNEL staining is quantified by counting the TUNEL-positive cells and then normalizing them to the total number of cells in each image. Three independent experiments were performed. Scale bar, 50 μm. The data are shown as the mean ± SD. *P <0.05, **P <0.01, ***P <0.001, and ****P <0.0001.

The House Ear Institute-Organ of Corti 1 (HEI-OC1) is one of the most common mouse auditory cell lines available for drug ototoxicity studies *in vitro* [19]. We exposed HEI-OC1 cells to different concentrations of cisplatin for 24 h to investigate its effects on their survival. We used flow cytometry to analyze dead and apoptotic cells, which were labeled with propidium iodide (PI) and Annexin V, respectively. As the cisplatin concentration increased, the proportions of both early and late apoptotic cells gradually increased (Fig. 1A–C, *P* <0.001, *n* = 3). Next, we applied TUNEL staining to further detect apoptotic HEI-OC1 cells after cisplatin treatment. The percentages of TUNEL-positive HEI-OC1 cells and total HEI-OC1 cells gradually increased as the cisplatin concentration increased (Fig. 1D, E, *P* <0.01, *n* = 3). Taken together, these results demonstrated that cisplatin significantly increases apoptosis of HEI-OC1 cells.

### The cGAS‑STING‑NF‑κB Pathway is Activated in HEI-OC1 Cells After Cisplatin Treatment

**Fig. 2:**
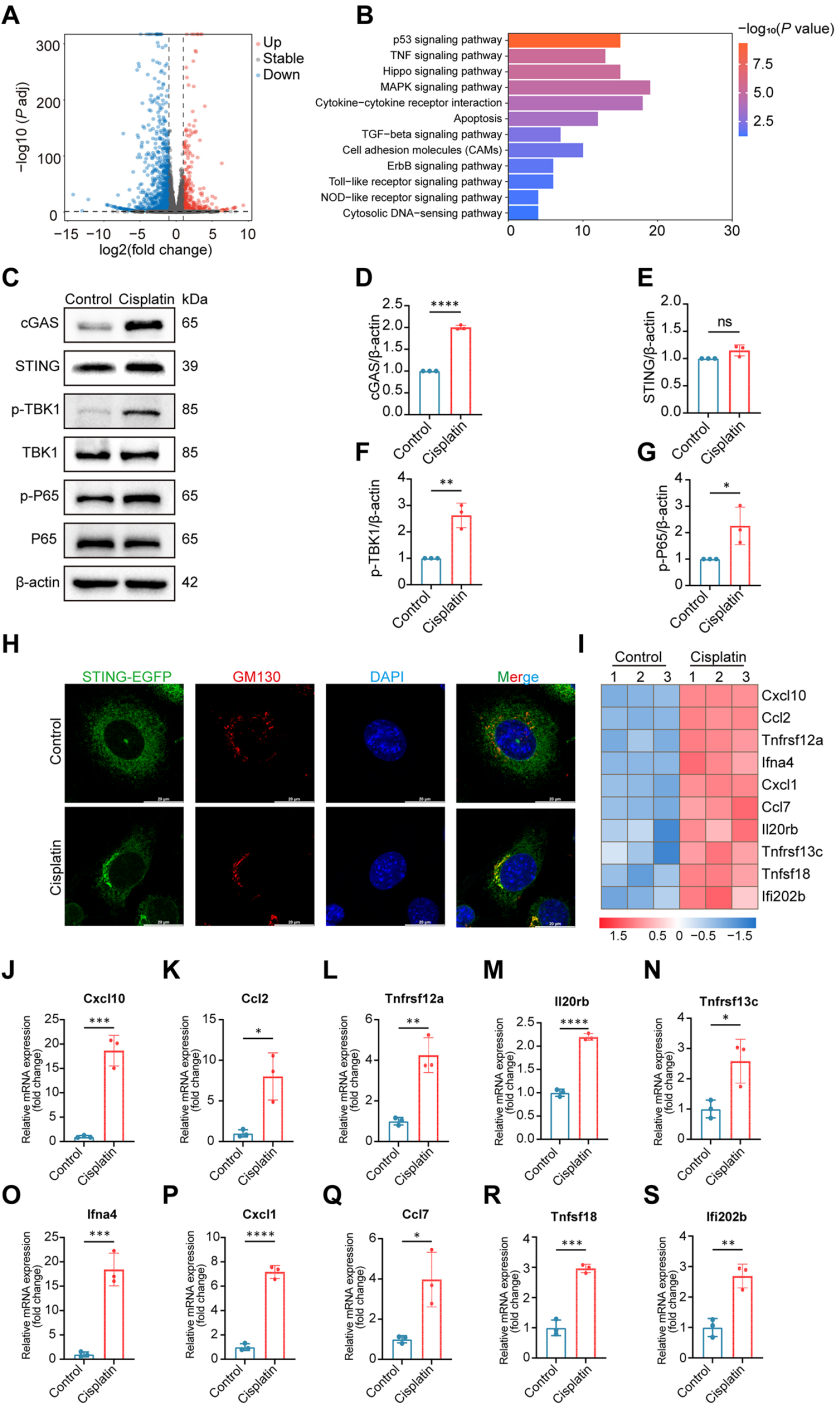
Cisplatin activates the cGAS-STING signaling pathway in HEI-OC1 cells. **A** Volcano plot showing dysregulated genes between the cisplatin and control groups. **B** KEGG-based gene function analysis showing the pathways most affected by cisplatin treatment in HEI-OC1 cells. **C**–**G** Western blots and quantification of cGAS, STING, p-TBK1, and p-p65 levels in the control and cisplatin groups (*n* = 3, unpaired *t* test). **H** HEI-OC1 cells expressing EGFP-tagged STING are stimulated with cisplatin. Representative images of immunofluorescence staining for STING (green), GM130 (red), and DAPI (blue) in HEI-OC1 cells. Scale bars, 20 μm. **I** Heatmap of differentially expressed proinflammatory genes in HEI-OC1 cells treated with or without cisplatin. **J**–**S** The relative mRNA levels of the indicated genes normalized to the β-actin level in HEI-OC1 cells treated with or without cisplatin, as determined by qRT‒PCR (*n* = 3, unpaired *t* test). The results are presented as the mean ± SD of three independent experiments. *P <0.05, **P <0.01, ***P <0.001, and ****P <0.0001.

To elucidate the mechanisms through which cisplatin reduces the viability of HEI-OC1 cells, RNA sequencing data from control and HEI-OC1 cells treated with 30 μmol/L cisplatin were analyzed to characterize the changes in gene expression. Our analysis revealed that exposure to cisplatin resulted in significant changes in the expression levels of thousands of genes. The RNA-seq results for the differentially-expressed genes between the control and cisplatin-treated cells are illustrated in volcano plots (Fig. 2A). We identified a total of 1874 genes that were differentially expressed between the control and cisplatin-treated cells, including 594 upregulated and 1280 downregulated genes. Further analysis of the upregulated genes using the Kyoto Encyclopedia of Genes and Genomes (KEGG) revealed genes associated with inflammation-related pathways, such as those for TNF signaling, cytokine‒cytokine receptor interaction, Toll-like receptor signaling, NOD-like signaling, and cytosolic DNA-sensing (Fig. 2B). The major genes of the inflammation pathway, including *Cxcl10, Ccl2, Tnfrsf12a, lfna4, Cxcl1, Ccl7, Il20rb, Tnfrsf13c, Tnfsf18,* and *lfi202b,* were markedly upregulated in HEI-OC1 cells after cisplatin treatment (Fig. 2I). Although inflammation is involved in cisplatin-induced ototoxicity, the innate immune pathways activated in HEI-OC1 cells after cisplatin treatment and their roles in HEI-OC1 cell injury are unclear. We subsequently measured the expression levels of genes associated with the cGAS-STING pathway and proinflammatory factors. We found that the cGAS-STING signaling pathway was strongly activated in the cisplatin group (Fig. 2C–G, *P* <0.05, *n* = 3). Because activated STING is transported to the Golgi, we investigated the colocalization of STING-EGFP with endogenous markers of the Golgi network in HEI-OC1 cells to further confirm that cisplatin activates the cGAS-STING pathway in these cells. Notably, compared with the control, cisplatin led to a significant increase in EGFP-STING localization with the Golgi signal (GM130) (Fig. 2H). In addition, we also found that the mRNA levels of proinflammatory factors were significantly increased in the cisplatin-treated HEI-OC1 cells (Fig. 2J–S, *P* <0.05, *n* = 3). These results strongly suggest that the cGAS-STING-NF-κB pathway is activated in HEI-OC1 cells after cisplatin exposure.

### Impaired Mitochondrial Function and mtDNA Release are Induced in HEI-OC1 Cells by Cisplatin

**Fig. 3:**
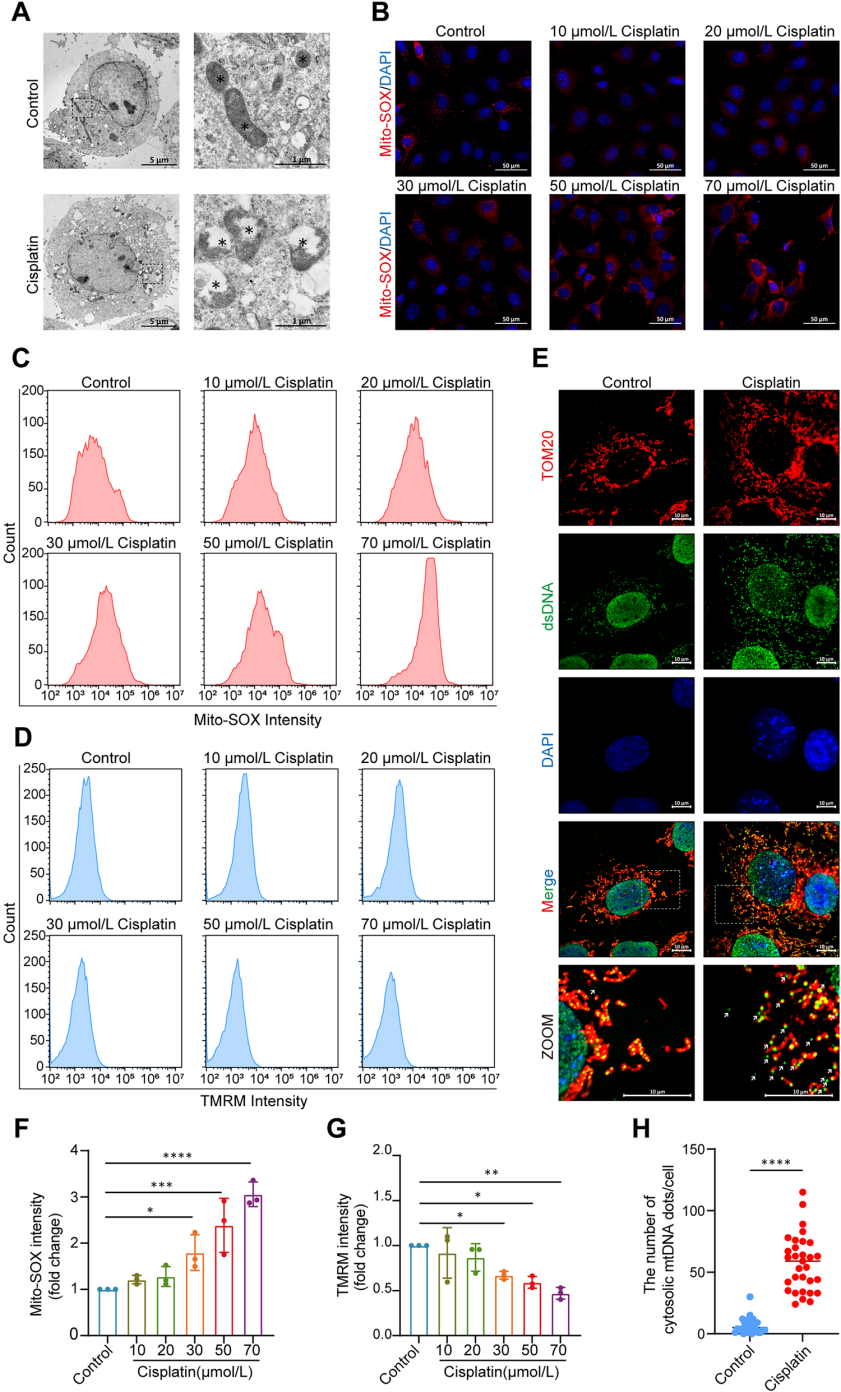
Impaired mitochondrial function and mtDNA release are induced by cisplatin treatment in HEI-OC1 cells. **A** Representative transmission electron microscopy images of each treatment group (asterisks: mitochondria). Scale bars, 5 μm. The magnified images on the right show the morphology of the mitochondria. Scale bars, 1 μm. **B** Representative images showing Mito-SOX fluorescence in HEI-OC1 cells from each group. Scale bars, 50 μm. **C** Flow cytometry analysis of Mito-SOX staining for mitochondrial ROS levels in each group (*n* = 3, ANOVA). **D** Flow cytometry analysis of TMRM staining for the mitochondrial membrane potential in each group (*n* = 3, ANOVA). **E** Representative superresolution AiryScan microscopy images of TOM20 (red), dsDNA (green), and DAPI (blue) in HEI-OC1 cells. Scale bars, 10 μm. Lower panel, enlarged representations of the mitochondria (red) and dsDNA (green), showing that DNA foci are located within the mitochondria, with some foci (arrowheads) in the cytoplasm of HEI-OC1 cells. **F** Changes in Mito-SOX fluorescence in the flow cytometry analysis of HEI-OC1 cells from each group (*n* = 3, ANOVA). **G** Changes in TMRM fluorescence in HEI-OC1 cells from each group (*n* = 3, ANOVA). **H** The numbers of cytosolic DNA dots/cell in E. Thirty cells were randomly selected for the statistical analysis (unpaired *t* test). The results are presented as the mean ± SD of three independent experiments. *P <0.05, **P <0.01, ***P <0.001, and ****P <0.0001.

Mitochondrial dysfunction has been suggested to be a hallmark of cisplatin-induced HC injury [22], and cytosolic DNA leakage from damaged mitochondria is a central activator of cGAS-STING signaling [23]. We therefore measured mitochondrial function in the cisplatin-treated HEI-OC1 cells. The transmission electron microscopy results revealed that the mitochondria in the untreated cells were normally shaped and had organized cristae, whereas, in the cisplatin-treated cells, the mitochondria were swollen, lacked orderly internal cristae, and formed cavities (Fig. 3A). These data confirmed that the mitochondria in HEI-OC1 cells were severely impaired after cisplatin treatment. We subsequently evaluated the levels of mitochondrial ROS and the mitochondrial membrane potential (MMP) to further assess the effects of cisplatin on mitochondrial function in these cells. First, we examined mitochondrial ROS production in cisplatin-treated HEI-OC1 cells using MitoSOX™ Red, an indicator of mitochondrial superoxide levels. Treatment with cisplatin gradually increased the fluorescence intensity of MitoSOX in the cells (Fig. 3B, C, F). We also used tetramethylrhodamine methyl ester (TMRM), a fluorescent indicator of the MMP [24], to examine its reduction after cisplatin treatment (Fig. 3D, G). We analyzed the cytosolic DNA content of HEI-OC1 cells using superresolution Airyscan imaging to establish whether mtDNA was released into the cytosol of cisplatin-treated cells. We applied coimmunostaining for TOM20, an outer membrane protein, and dsDNA to assess mtDNA release. Interestingly, we found a prominent cytosolic accumulation of mtDNA in the mitochondria, which was largely absent in the control group (Fig. 3E, H). Collectively, these results indicated that cisplatin induces mitochondrial dysfunction and mtDNA leakage.

### Knocking Down STING Blocks Cisplatin‑induced Inflammation and Apoptosis in HEI-OC1 Cells

**Fig. 4:**
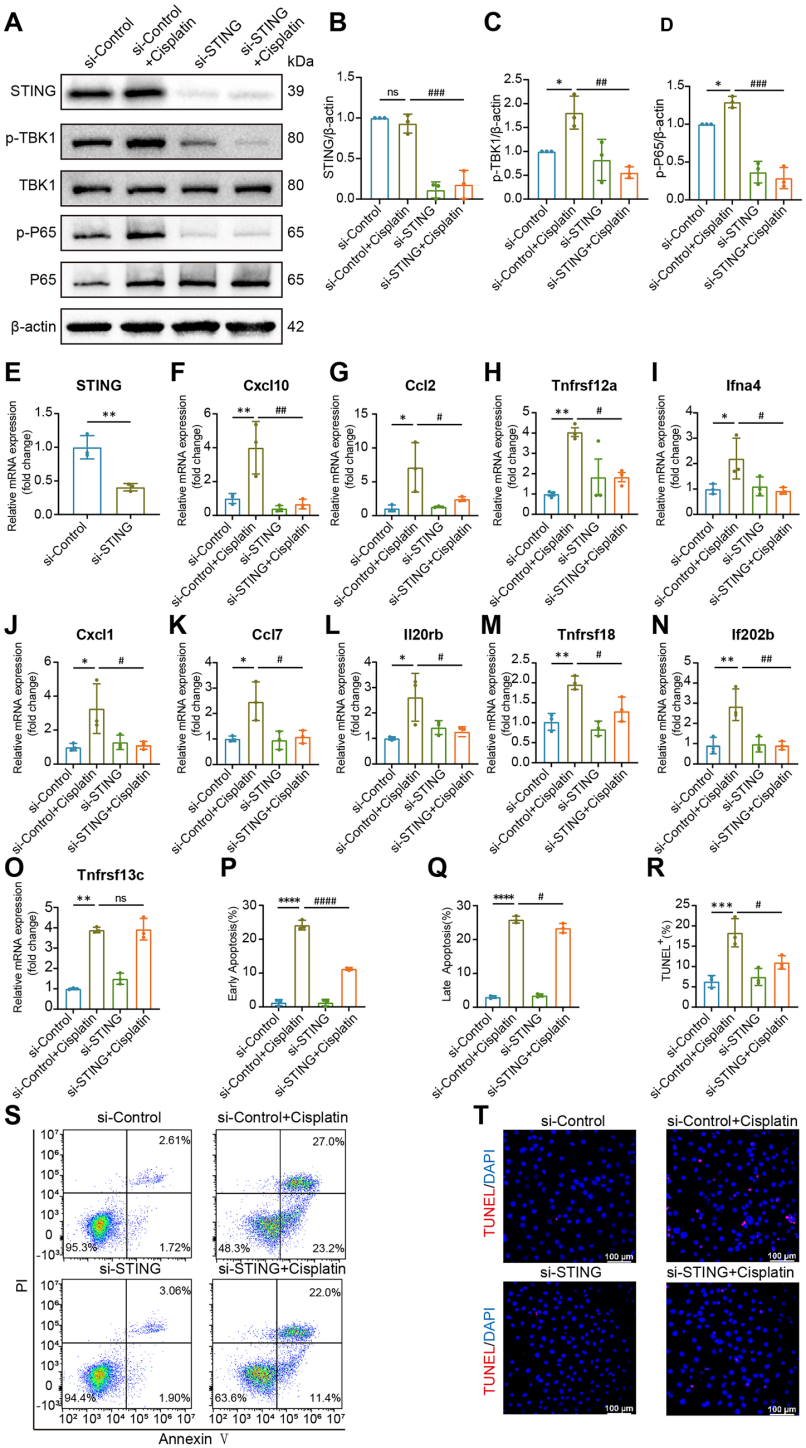
Knocking down STING decreases cisplatin‑induced inflammation and damage in HEI-OC1 cells. **A**–**D** Western blots and quantification of cGAS, STING, p-TBK1, and p-p65 levels in the control and cisplatin-treated cells from each treatment group (*n* = 3, ANOVA). **E**–**O** The relative mRNA levels of the indicated genes normalized to the β-actin level in HEI-OC1 cells from each treatment group, as determined by qRT‒PCR (*n* = 3, ANOVA). **P** The early apoptosis ratio in S (*n* = 3, ANOVA). **Q** The late apoptosis ratio in S (*n* = 3, ANOVA). **R** Percentages of TUNEL-positive cells (*n* = 3, ANOVA). TUNEL staining was quantified by counting the TUNEL-positive cells and then normalizing them to the total number of cells in each image. **S** The flow cytometry analysis of each treatment group using an Annexin V-FITC/PI kit. **T** Representative images of TUNEL staining in HEI-OC1 cells. Scale bars, 100 μm. The results are presented as the mean ± SD of three independent experiments. *P <0.05, **P <0.01, ***P <0.001, and ****P <0.0001 *versus* control; ^#^*P* <0.05, ^##^*P* <0.01, ^###^*P* <0.001, and ^####^*P* <0.0001 *vs* si-Control + cisplatin.

To further assess the specific function of the cGAS-STING pathway in cisplatin-induced ototoxicity *in vitro*, we used small interfering RNA (siRNA) to knock down STING in HEI-OC1 cells. Cisplatin (30 μmol/L) was used to treat HEI-OC1 cells transfected with either the negative control siRNA (si-Control) or STING siRNA (si-STING). Western blot analysis showed that transfecting HEI-OC1 cells with si-STING significantly downregulated STING expression and significantly diminished the cisplatin-induced phosphorylation of TBK1 and the NF-κB p65 subunit (Fig. 4A–D). We also found that the expression of most inflammatory factors was downregulated after STING knockdown (Fig. 4F–R).

We used flow cytometry and TUNEL staining to determine the effect of STING knockdown on HEI-OC1 cell survival. Compared with those among control cells, the proportions of apoptotic cells were dramatically higher in the cisplatin group. However, si-STING combined with cisplatin decreased these proportions (Fig. 4P, Q, S). The confocal microscopy results revealed that cells treated with cisplatin had stronger TUNEL signals than those in the si-Control group, but the si-STING+cisplatin group had fewer TUNEL-positive cells than the cisplatin group (Fig. 4R, T). Taken together, the Annexin/PI and TUNEL staining results showed that cisplatin-induced apoptosis was alleviated by the inhibition of STING. In addition, we used the STING-specific small molecule H-151 to treat HEI-OC1 cells to further determine whether inhibiting STING activation can inhibit cisplatin-induced damage to HEI-OC1 cells. Similar to the results of the knockdown experiments, H-151 reduced the cisplatin-induced apoptosis (Supplementary Fig. 1A–E).

### Inhibition of STING Promotes Cochlear HC Survival after Cisplatin-induced Damage

**Fig. 5:**
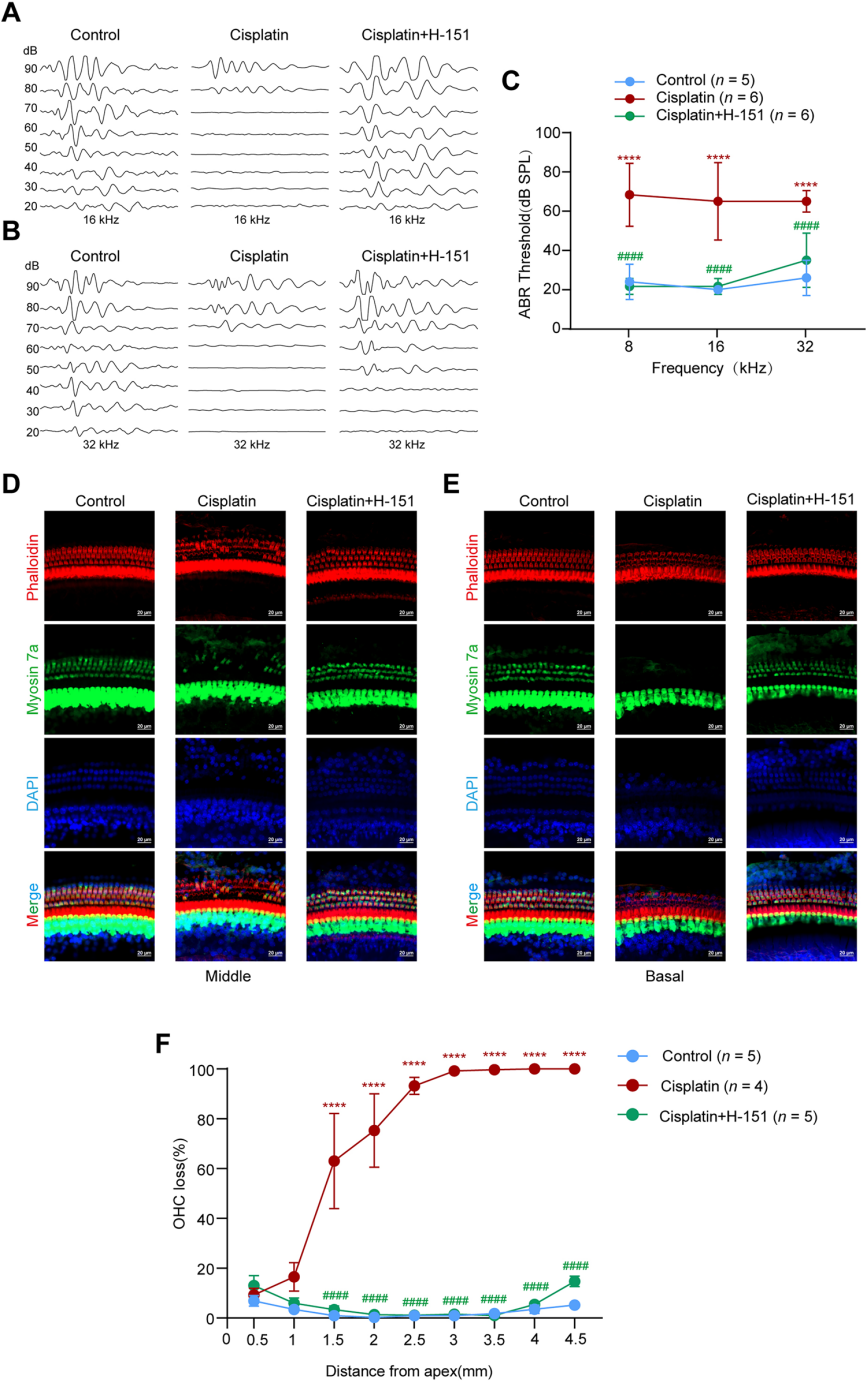
Effects of H-151 on cisplatin-induced hearing loss *in vivo*. **A** Representative tone burst ABR images at 16 kHz for each group. **B** Representative tone burst ABR images at 32 kHz for each group. **C** ABR thresholds after exposure to cisplatin with or without H-151 (ANOVA). Control (sterile saline only): *n* = 5; cisplatin (2 mg/kg cisplatin+200 mg/kg furosemide): *n* = 6; cisplatin+H-151 (10 mg/kg H-151+2 mg/kg cisplatin+200 mg/kg furosemide): *n* = 6. **D** Immunofluorescence staining for myosin 7a (green) and phalloidin (red) in the middle turns of the cochleae from different groups. Scale bars, 20 μm. **E** Immunofluorescence staining for myosin 7a (green) and phalloidin (red) in the basal turns of the cochleae from different groups. Scale bars, 20 μm. Control ( sterile saline only): *n* = 5; cisplatin (2 mg/kg cisplatin+200 mg/kg furosemide): *n* = 4; cisplatin+H-151 (10 mg/kg H-151+2 mg/kg cisplatin+200 mg/kg furosemide): *n* = 5. **F** The OHC death ratio in each group (ANOVA). *P <0.05, **P <0.01, ***P <0.001, and ****P <0.0001 *vs* the control group; ^#^*P* <0.05, ^##^*P* <0.01, ^###^*P* <0.001, and ^####^*P* <0.0001 *vs* the cisplatin group. OHCs, outer hair cells.

Given that cisplatin can trigger the cGAS-STING pathway *in vitro*, we investigated whether inhibiting STING activity with H-151, a selective small-molecule STING inhibitor [25] that inhibits STING palmitoylation, could exert protective effects on HC injury *in vivo*. Mouse inner ear tissue seems to be strongly resistant to cisplatin. Scholars have speculated that the blood–labyrinth barrier in the inner ear of mice may be one of the important reasons for the resistance of the inner ear tissue to ototoxic drugs [26]. According to a previous report, furosemide can temporarily disrupt the stria vascularis and open the blood‒ear barrier, allowing the entry of cisplatin into the inner ear [16]. Therefore, we constructed a model of cisplatin-induced ototoxicity in mice *via* the co-administration of furosemide and cisplatin. In the cisplatin group, we administered 200 mg/kg furosemide and 2 mg/kg cisplatin daily for three consecutive days to FVB/NJ mice. In the cisplatin + H-151 group, FVB/NJ mice were administered H-151 at 10 mg/kg body weight one day before the first cisplatin treatment. The next day, 1 h before cisplatin treatment, the mice were injected with furosemide and then with 2 mg/kg cisplatin. The mice in the cisplatin + H-151 group were treated with furosemide and cisplatin for three consecutive days. Saline-treated mice that were not given cisplatin constituted the control group. The ABR was determined 3 days later. The ABR results revealed that cisplatin + furosemide administration significantly elevated the ABR threshold, whereas the H-151 injection significantly reduced the elevated threshold (Fig. 5A–C). The cochleae were subsequently harvested, and the HCs were labeled with phalloidin and myosin 7a. Compared with the control group, the cisplatin group presented extensive loss of OHCs in both the basal and middle cochlear sections and pretreatment with H-151 markedly reduced the OHC loss (Fig. 5D–F). Taken together, these findings suggest that pharmacological STING inhibition with H-151 can improve the function and restore the morphology of HCs after cisplatin exposure, highlighting the potential benefits of preventing cisplatin-induced hearing loss.

## Discussion

Cisplatin is a widely used chemotherapeutic drug [27]. However, progressive and permanent hearing loss is a serious side-effect in patients receiving this treatment. Reducing this ototoxicity is a key focus of hearing research. Inflammation is considered to play an important role in the pathological mechanism of cisplatin-induced ototoxicity [28]. However, whether the cGAS-STING-NF-κB axis is involved in the inflammatory response of cochlear HCs following cisplatin treatment remains to be clarified. Our in-depth study verified that cisplatin significantly activated the cGAS-STING pathway and that inhibition of this pathway reduced inflammatory factor expression and HC damage to a certain extent. To our knowledge, this report is the first to show that the cGAS-STING-NF-κB pathway is activated in HEI-OC1 cells after cisplatin injury and induces the production of downstream inflammatory factors. This study also revealed that the pharmacological inhibitor H-151 has a protective effect on cochlear HCs *in vivo*. These results demonstrated that inhibiting the cGAS-STING-NF-κB pathway could be a novel therapeutic strategy for cisplatin-induced ototoxicity.

Inflammation may be an early event in cisplatin-induced injury. In response to cisplatin, HEI-OC1 cells produce proinflammatory factors, such as TNF-α, IL-1β, IL-6, and NF-κB [29]. Innate immune pathways participate in the inflammatory response and are not only activated by pathogens but also activated after damage-associated molecular patterns are detected after injury or during aseptic inflammation [30]. The Toll-like receptor (TLR) signaling pathway is the most thoroughly studied innate immune pathway involved in cisplatin-induced ototoxicity. An *in vitro* experiment showed that cisplatin can activate TLR4, a membrane‐bound pattern recognition receptor, which then upregulates the MyD88‐dependent signaling components NF‐κB and p42/44 and promotes inflammation and cell death in HCs [31]. In this study, we found that the cGAS-STING pathway, another pathway involved in the innate immune system, and its downstream target NF-κB were activated and ultimately upregulated the expression of inflammatory factors in HEI-OC1 cells after cisplatin injury. The cytoplasmic DNA biosensor cGAS can be activated in the presence of cytosolic dsDNA, converting ATP and GTP into the second messenger cGAMP [32]^.^ This process initiates the activation of STING. Next, activated STING phosphorylates NF-κB *via* TBK1 [33]. This sequence of events triggers a sterile inflammatory response, worsening tissue damage [34–36]. More recently, the cGAS-STING pathway was shown to mediate interferon I signaling and the inflammatory response in the pathogenesis of age-related hearing loss [37]^.^ Melatonin inhibits the cGAS-STING signaling pathway and reduces the expression of cytokines in the cochlea, partially protecting against hearing loss in a presbycusis mouse model [38]^.^ However, little is known about the correlation between the cGAS-STING pathway and cisplatin-induced hearing loss. In the present study, we found increased levels of phosphorylated TBK1 and p65 in HEI-OC1 cells treated with cisplatin, accompanied by increased expression of proinflammatory factors, whereas compared with those in the cisplatin-only group, cisplatin-induced injury was reduced by siRNA-mediated STING knockdown, with decreased expression of proinflammatory cytokines in HEI-OC1 cells. Notably, the injection of a STING inhibitor blocked cisplatin-induced cell death in mice. Thus, the above findings confirmed that cGAS-STING signaling is a harmful regulator of the response to cisplatin exposure.

Inflammation is also closely associated with apoptosis. Previous studies have reported that apoptosis, a type of programmed cell death, in HCs, is the mechanism underlying cisplatin-induced ototoxicity [39, 40]^.^ Consistent with previous studies, we found that the ratios of apoptotic/total HEI-OC1 cells and TUNEL-positive/total HEI-OC1 cells were significantly increased in response to cisplatin-induced injury, indicating that cisplatin can cause the death of HCs mainly by activating apoptosis. The cGAS-STING pathway has been shown to regulate apoptosis. Gulen *et al.* [41] reported that the activation of STING induces apoptosis in CD4+ T cells. STING overexpression promotes the expression of the apoptotic markers BAX, Cyto-C, and cleaved caspase-3, and STING knockdown also attenuates IL-1β-induced chondrocyte apoptosis [42]. Interestingly, fewer apoptotic cells and fewer TUNEL-stained HEI-OC1 cells were found in the STING knockdown group than in the control group, suggesting that STING inhibition protects HCs from cisplatin-mediated ototoxicity through the inhibition of apoptosis. As noted in this study, cisplatin-induced apoptosis may involve inflammatory pathways. Inflammation seems to be an early event in cochlear cells after cisplatin treatment, and more studies are needed to determine the specific molecular mechanisms of inflammation and apoptosis. Notably, inhibiting STING had a much greater effect on hair cells *in vivo* than *in vitro*, which may be due to the involvement of immune cells. In a mouse ototoxicity model, monocyte infiltration and macrophage activation occur in damaged areas of the basilar membrane and spiral ganglion cells [43]. In addition, sustained depletion of macrophages by PLX3397 completely prevents cisplatin-induced hearing loss and OHC dysfunction in a mouse model [44]. In our study, some chemokines were upregulated in HEI-OC1 cells after cisplatin treatment, and these may be signals for the recruitment of cochlear macrophages to the damaged area for a local inflammatory response. Therefore, we speculate that macrophages may play an important role in cisplatin-induced hearing loss, and we plan to conduct follow-up studies on the specific mechanism of action involved.

Mitochondrial dysfunction is considered an important mechanism in the pathological process of HC damage [45, 46]^.^ Here, we visually observed mitochondrial damage *via* transmission electron microscopy. Overproduction of ROS overwhelms the redox balance, triggering mitochondrial depolarization and subsequent apoptosis of hair cells [7]. In the present study, we used MitoSOX, a mitochondria-specific ROS indicator, to determine whether cisplatin could increase ROS generation. We also used TMRM dye to determine the decrease in the MMP after cisplatin treatment. Recent studies have indicated that impaired mitochondria potentially drive detrimental inflammatory responses through the release of mtDNA [47]^.^ Released mtDNA serves as an internal trigger for cGAS, thereby activating the innate immune system *via* the cGAS-STING pathway [48]^.^ Here, we verified cGAS-STING pathway activation and increased mtDNA release in cisplatin-treated HEI-OC1 cells. mtDNA may be the ligand activating the cGAS-STING pathway in cisplatin-induced ototoxicity. The limitation of this study is that it preliminarily showed that cytosolic mitochondrial DNA may be the activation signal for the cGAS-STING pathway in HEI-OC1 cells, but how mtDNA escapes from mitochondria in HEI-OC1 cells is still unclear. We plan to conduct in-depth research on this topic in the future.

**Fig. 6:**
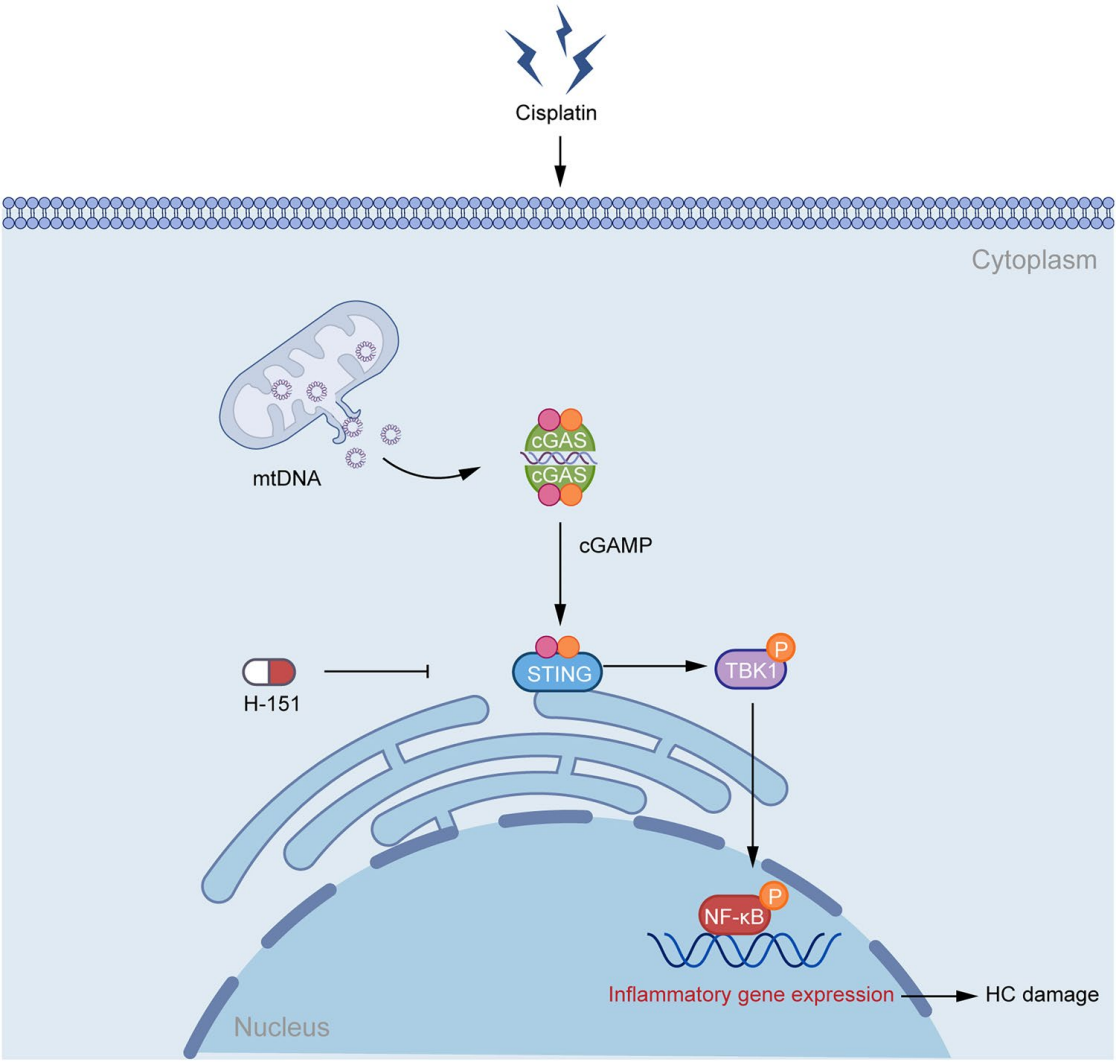
Mechanism of the cGAS-STING pathway in cisplatin-induced HC damage.

Overall, we showed that the cGAS-STING pathway is important for the activation of proinflammatory signaling leading to cisplatin-induced ototoxicity. The inhibition of STING attenuated inflammatory reactions, protected against cisplatin-induced ototoxic damage, and improved hearing function after cisplatin administration (Fig. 6). These findings indicate that STING might be a therapeutic target for cisplatin-induced hearing loss.

## Supplementary Information

Below is the link to the electronic supplementary material.

Supplementary file1 (PDF 242 kb)

## Data Availability

The sequencing data reported in this paper have been deposited in SRA (PRJNA1080024) and are publicly available.
