# Supplementary material for: Inhibition of the cGAS‑STING Pathway Reduces Cisplatin-Induced Inner Ear Hair Cell Damage
Source: Neurosci Bull. 2024 Dec 16;41(3):359–73. doi: 10.1007/s12264-024-01334-8 (PMC11876498; doi:10.1007/s12264-024-01334-8)
Supplement: Supplementary file 1 — Supplementary file1 (PDF 242 kb) [file 12264_2024_1334_MOESM1_ESM.pdf]

Supplementary Figure and Figure Legend

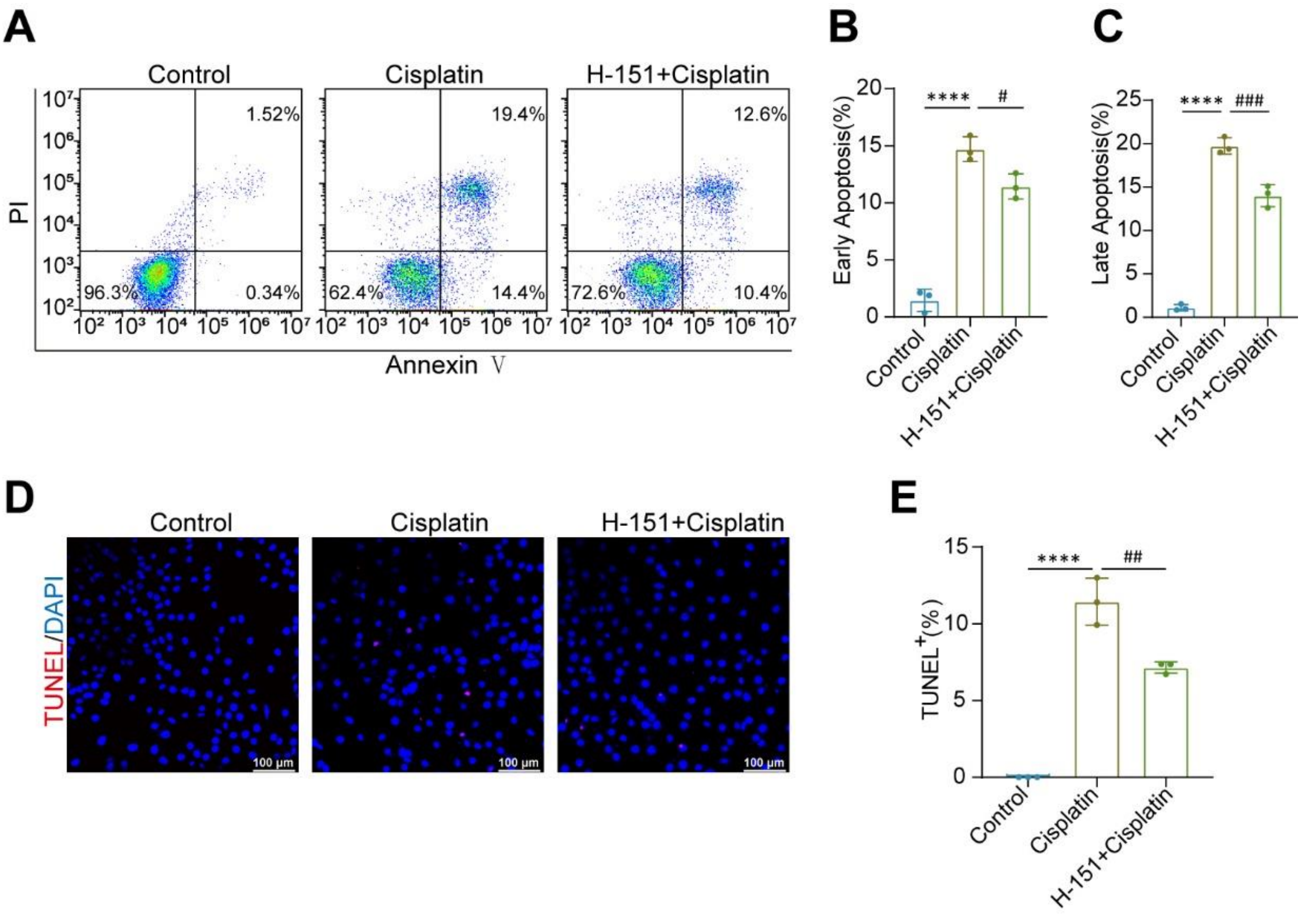

Fig. S1 H-151 decreases cisplatin-induced damage in HEI-OC1 cells. **A** Flow cytometry analysis of each treatment group using an Annexin V-FITC/PI kit. HEI-OC1 cells were treated with 10 μmol/L H-151 and 30 μmol/L cisplatin for 24 h. **B** The late apoptosis ratio in A (*n* = 3, ANOVA). **C** The early apoptosis ratio in A (*n* = 3, ANOVA). **D** Representative images of TUNEL staining in HEI-OC1 cells. Scale bars, 100 μm. HEI-OC1 cells were treated with 10 μmol/L H-151 and 30 μmol/L cisplatin for 24 h. **E** TUNEL staining quantified by counting the TUNEL-positive cells and then normalizing to the total number of cells in each image (*n* = 3, ANOVA). The all results are presented as the mean ± SD of three independent experiments. ****P <0.0001 *vs* the control, $^{\#}P$ <0.05, $^{\#\#}P$ <0.01, and $^{\#\#\#}P$ <0.001 *vs* cisplatin.
